# Immunophenotyping of peripheral blood cells allows to discriminate MIS-C and Kawasaki disease

**DOI:** 10.1186/s41231-022-00128-2

**Published:** 2022-09-04

**Authors:** Alice Castaldo, Carolina D’Anna, Monica Gelzo, Antonietta Giannattasio, Marco Maglione, Stefania Muzzica, Maddalena Raia, Giulia Scalia, Lorella Tripodi, Giuseppe Castaldo, Vincenzo Tipo, Domenico Grieco, Michela Grieco

**Affiliations:** 1 Dipartimento Di Scienze Mediche Traslazionali, Sezione Di Pediatria, Università Di Napoli Federico II, Naples, Italy; 2 Dipartimento Di Emergenza, AORN Santobono-Pausilipon, Naples, Italy; 3 CEINGE-Biotecnologie Avanzate, via Gaetano Salvatore 486, 80145, scarl, Naples, Italy; 4 Dipartimento Di Medicina Molecolare E Biotecnologie Mediche, Università Di Napoli Federico II, Naples, Italy

**Keywords:** MIS-C, Kawasaki disease, Flow cytometry

## Abstract

**Background:**

The pathogenesis of the novel described multisystem inflammatory syndrome in children (MIS-C) and Kawasaki disease (KD) is still debated as it is not clear if they are the same or different nosological entities. However, for both the diseases a rapid and unequivocal diagnosis is mandatory to start the therapy before the onset of severe complications. In this study, we aimed to evaluate the white cell populations in MIS-C and KD as potential markers to discriminate between the two diseases.

**Methods:**

We studied white cell populations by flow cytometry in 46 MIS-C and 28 KD patients in comparison to 70 age-matched healthy children.

**Results:**

MIS-C patients had a significant lymphopenia that involved both B and T populations while KD patients showed a significant neutrophilia and thrombocythemia. Granulocyte/lymphocyte ratio helped to diagnose both MIS-C and KD with a high diagnostic sensitivity, while a multivariate analysis of granulocyte and T lymphocyte number contributed to discriminate between the two diseases.

**Conclusions:**

The relevant lymphopenia observed in MIS-C patients suggests that the disease would be a post-infectious sequel of COVID-19 immunologically amplified by a massive cytokine release, while the significant neutrophilia and thrombocythemia observed in KD confirmed that the disorder has the genesis of a systemic vasculitis. The analysis of a panel of circulating cells may help to early diagnose and to discriminate between the two diseases.

**Supplementary Information:**

The online version contains supplementary material available at 10.1186/s41231-022-00128-2.

## Introduction

A putatively novel entity called multisystem inflammatory syndrome in children (MIS-C) by the US Centre for Disease Control and Prevention has been recently described [1]. An epidemiological study in Italy found an incidence rate of 10.3/100 000 residents with an age < 19 years from March 2020 to June 2021 [2]. The novel syndrome was initially considered an atypical form of Kawasaki disease (KD) because it appears with fever, vasculitis, conjunctival and mucocutaneous involvement and may result in cardiac complications. However, the appearance of left ventricular dysfunction and cardiovascular shock, coagulopathy, and gastrointestinal involvement, frequent in patients with MIS-C [3] and rarely observed in KD, and the different epidemiology (i.e., KD typically occurs before 5 years of age with an incidence rate of 17.6/100,000 children [4], while MIS-C frequently affects elder children and adolescents) suggested the novel syndrome may have a different pathogenesis. In fact, MIS-C usually appears 3–6 weeks after Sars-CoV-2 infection and likely represents a hyperimmune, delayed response to SARS-CoV-2 virus that resemble the adult, severe infection [5]. In children, an early mild/asymptomatic infection with SARS-CoV-2 seems to trigger macrophage activation followed by the stimulation of T-helper cells. This event causes the cytokine release, and the consequent stimulation of macrophages, neutrophils, and monocytes together with plasma cell activation leading a hyperimmune response that is associated with the inflammatory syndrome [6]. While, KD may arise by a still unknown ubiquitous agent that usually causes an asymptomatic infection infecting bronchial epithelial cells but led to KD in genetically predisposed children. The agent may persist in cytoplasmic inclusion bodies, with intermittent shedding after acute infection. The enhancement of cytotoxic T cell and interferon pathway expression in the coronary arteries of not survived KD children strongly suggest a viral etiology. In addition, it has been observed the production of self-reactive antibodies with the large involvement of IgA producing plasma cells [7]. Subsequently, neutrophils infiltrate arteries causing a necrotizing arteritis [8].

Various studies described and compared cellular alterations found in patients with MIS-C and with KD with different results. Leukopenia and a more severe lymphopenia were described in patients with MIS-C [9]; another study reported lymphopenia in MIS-C and neutrophilia in KD [6]. The neutrophil–lymphocyte ratio was suggested as a predictor of KD severity since a significant neutrophilia and lymphopenia were observed in severe KD [10, 11]. The deep immune profiling of patients with MIS-C demonstrated a marked, although transient, lymphopenia with a more pronounced T-cell involvement i.e., the same profile that we observed in adult patients with severe, acute Sars-CoV-2 infection [12, 13]. Furthermore, a suppression of CD8 was reported in KD [14]. The circulating cell profile of patients with the two diseases helps to define diagnostic biomarkers that may contribute to the early diagnosis of each of the two entities and also to discriminate between them [15] since different outcome and therapy response were reported in patients with KD and MIS-C [16].

For these reasons we studied white cell populations in 46 patients with MIS-C and in 28 patients with KD (these latter diagnosed between 2017 and 2019, before the Sars-CoV-2 pandemic), and for each group we compared the data with those obtained in a control population of 70 healthy children to the aim of developing a biomarker model useful to discriminate between MIS-C and KD.

## Methods

### Patients

The study was approved by the Ethical Committee of the University Federico II of Naples. We studied 46 patients with the diagnosis of MIS-C (median age: 7 years, range: 1–14 years, 17 females) and 28 with the diagnosis of KD (median age: 2 years, range: 1–4 years, 19 females) at hospital admission. The patients with MIS-C were admitted between June 2021 and April 2022, while those with KD had been admitted between 2017 and 2019, before the Sars-CoV-2 pandemic. The diagnosis of KD was performed according to the American Heart Association [17]. The diagnosis of MIS-C was performed using the case definition criteria of the CDC Health Alert Network [1]. Furthermore, we studied a control group of 70 healthy children with an interval of age matching both MIS-C and KD patients (median age: 4 years, range 1–14 years, 14 females). The number of males resulted significant different among the MIS-C and KD patients and controls (MIS-C *versus* controls, p = 0.043; KD *versus* controls, *p* < 0.0001; KD *versus* MIS-C, *p* = 0.01).

### Immunophenotyping of peripheral blood cells

Whole blood samples were collected at admission from in tubes containing EDTA. White blood cell count (WBC) and platelets count were assayed by a blood cell counter Sysmex SF-3000 cell counter (Dasit SpA, Cornaredo, MI, Italy). Cytometric analysis was performed by multicolour flow cytometry as previously described [12]. Briefly, lymphocytes, granulocytes, and monocytes were firstly separated on the basis of forward scatter and sideward scatter characteristics. Lymphocyte cells were gated by using CD45, and this gate was used to identify the T helper (TH) cells (CD3 + and CD4 +) and T cytotoxic (CD3 + and CD8 +) lymphocytes. (Figure S1-A, Additional file 1). CD19 was used to identify B cells (Figure S1-B, Additional file 1). The values have been expressed both as percentage and absolute numbers.

### Statistical analysis

Statistical differences between the three groups were assessed by Kruskal–Wallis test and Mann–Whitney U test as post-hoc test. The chi-square test was used to compare the frequency of males/female between the groups. To test the association between white cell parameters and gender, a linear regression analysis with male gender as independent variable was performed using a stepwise approach. Principal Component Analysis (PCA) was applied to detect sample metabolite trends and clustering in an unsupervised manner, and the Partial Least-Squares Discriminant Analysis (PLS-DA) was then performed to reinforce classification and to better identify clustering. Before PCA and PLS-DA, data were batch normalized dividing each variable of each group by the median of all original values of that group. Finally, the dataset was square root transformed and auto-scaled. On the base of the VIP score obtained from PLS-DA analysis, we selected the variables (top features, n = 5) for the receiver operating characteristic (ROC) analysis. In fact, the VIP score is a measure of the importance of a variable in the PLS-DA model and expresses the contribution of a variable to a biomarker model. The ROC curve based exploratory analysis (Explorer) was used and areas under the curve (AUC) were calculated to compare the effectiveness of the top features to discriminate the first 23 MIS-C from the 28 KD patients. According to the criteria of Jones and Athanasiou [18], AUC > 0.97, 0.93–0.96, 0.75–0.92, and 0.6–0.74 were interpreted as “excellent,” “very good,” “good,” and “reasonable,” respectively. Successively, we selected the n feature-model with the higher AUC and prediction accuracy and applied it in the ROC curve based model evaluation (Tester). For the model validation, we applied this model also for the class prediction of an independent subset of new MIS-C samples (*n* = 23). For both ROC Explorer and Tester analyses, we selected the linear support vector machines (SVM) classification, as a supervised machine learning algorithm, where each pattern belongs to a predefined class, and SVM built-in as feature ranking method. Statistical analysis was performed by SPSS (version 27, IBM SPSS Statistics) and MetaboAnalyst 5.0 online package [https://www.metaboanalyst.ca]. Graphics have been performed by KaleidaGraph software (version 4.5.4, Synergy, Reading, PA, USA). *P* values < 0.05 were considered as significant.

## Results

### Immunophenotyping of peripheral blood cells

Figure S2-A (see Additional file 1) shows total blood leukocytes (WBC, reported as absolute number/mmc) in patients with MIS-C and in patients with KD, compared with the control group. Either in patients with MIS-C and in patients with KD the number of leukocytes was significantly higher than that observed in the control group; furthermore, the number of leukocytes in patients with KD was significantly higher of that observed in patients with MIS-C. Figure S2-B (see Additional file 1) shows the number of platelets (reported as number × 10^3^/mmc) in patients with MIS-C and in patients with KD, compared with the control group. Patients with MIS-C had a number of platelets significantly lower as compared both to controls and to patients with KD. These latter had a number of platelets significantly higher as compared to control subjects.

Figure S3 (see Additional file 1) shows the data of total blood granulocytes, lymphocytes and monocytes, each reported as percentage of total leukocytes and as absolute number/mmc in patients with MIS-C and in patients with KD, compared with the control group. Both patients with MIS-C and patients with KD had a significantly higher percentage of granulocytes, a significantly lower percentage of lymphocytes and a not significantly different percentage of monocytes as compared to control subjects. In MIS-C patients we found a higher percentage of granulocytes and a lower percentage of lymphocytes as compared to KD patients. In addition, the number of granulocytes resulted significantly higher in both the groups of patients as compared to the control group and significantly higher in patients with KD as compared to patients with MIS-C. Furthermore, patients with MIS-C had a significantly lower number of lymphocytes as compared either to the control group and as compared to patients with KD. In these latter patients, the number of lymphocytes was higher than the control group. Finally, patients with KD had a significantly higher number of monocytes as compared both to the control group and to patients with MIS-C.

Figure S4 (see Additional file 1) shows the data of total T, T helper and T cytotoxic lymphocytes each reported as percentage of total lymphocytes and as absolute number/mmc in patients with MIS-C and in patients with KD, compared with the control group. Both patients with MIS-C and patients with KD had a significantly lower percentage of total T, T helper and T cytotoxic lymphocytes as compared to the control group. Furthermore, patients with MIS-C had a significantly higher percentage of T lymphocytes and a significantly lower percentage of T cytotoxic as compared to patients with KD, while no differences were observed between the two groups of patients for T helper lymphocytes. Both patients with MIS-C and patients with KD had a significantly lower number of T lymphocytes as compared to the control group; furthermore, patients with MIS-C had a significantly lower number of T lymphocytes as compared to patients with KD. Patients with MIS-C had a significantly lower number of either T helper and T cytotoxic lymphocytes as compared both to controls and to patients with KD. In these latter, the number of both T helper and T cytotoxic lymphocytes was not significantly different as compared to the control group.

Figure S5 (see Additional file 1) shows the data of total B lymphocytes reported as percentage of total lymphocytes and as absolute number in patients with MIS-C and in patients with KD, compared with the control group. Patients with MIS-C had a significantly higher percentage of B lymphocytes as compared both to the control group and to patients with KD. These latter had a significantly lower percentage of B lymphocytes as compared to the control group. While, patients with MIS-C had a significantly lower number of B lymphocytes as compared both to the patients with KD and to the control group.

**Fig. 1:**
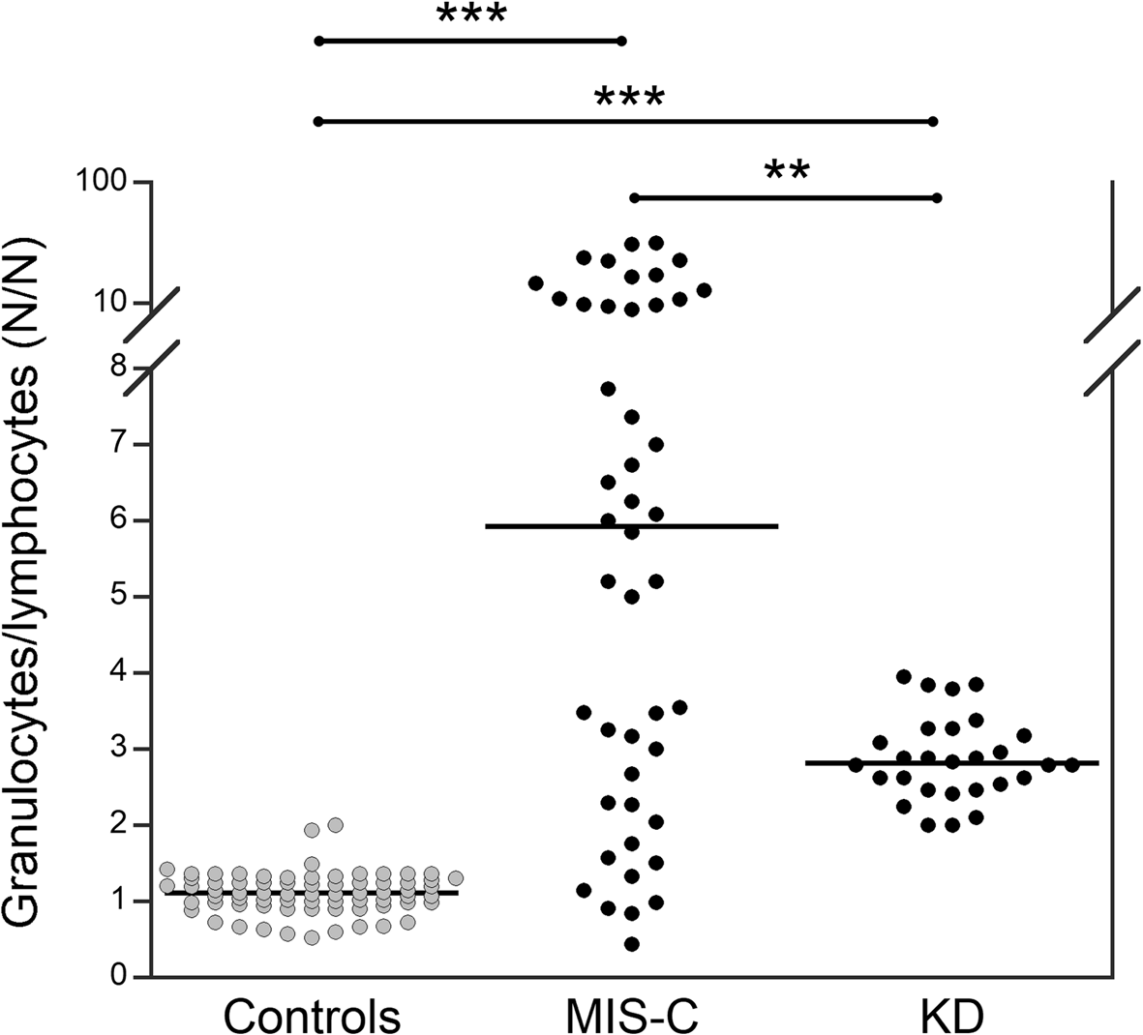
Comparison of granulocytes/lymphocytes ratio in controls (*n* = 70), MIS-C (*n* = 46) and KD (*n* = 28) patients at hospital admission. *p < 0.0001. KD: Kawasaki disease; MIS-C: multisystem inflammatory syndrome in children

As shown in Fig. 1, the granulocyte/lymphocyte ratio was significantly higher in patients with MIS-C as compared both to the control group and patients with KD, and these latter had a significantly higher ratio as compared to the controls. Such ratio, at a cut-off value of 1.75 had also a satisfactory diagnostic efficiency in discriminating both MIS-C (diagnostic sensitivity: 78.2%) and KD (diagnostic sensitivity: 96.4%) from healthy subjects (diagnostic specificity: 97.2%).

The regression analysis did not reveal a significant effect of male gender on the percentage and number of white cells, except for the percentage (slope: -0.336, *p*: 0.040) and number (slope: -0.323, *p*: 0.047) of B lymphocytes in patients with KD.

### PCA, PLS-DA and ROC analyses

**Fig. 2:**
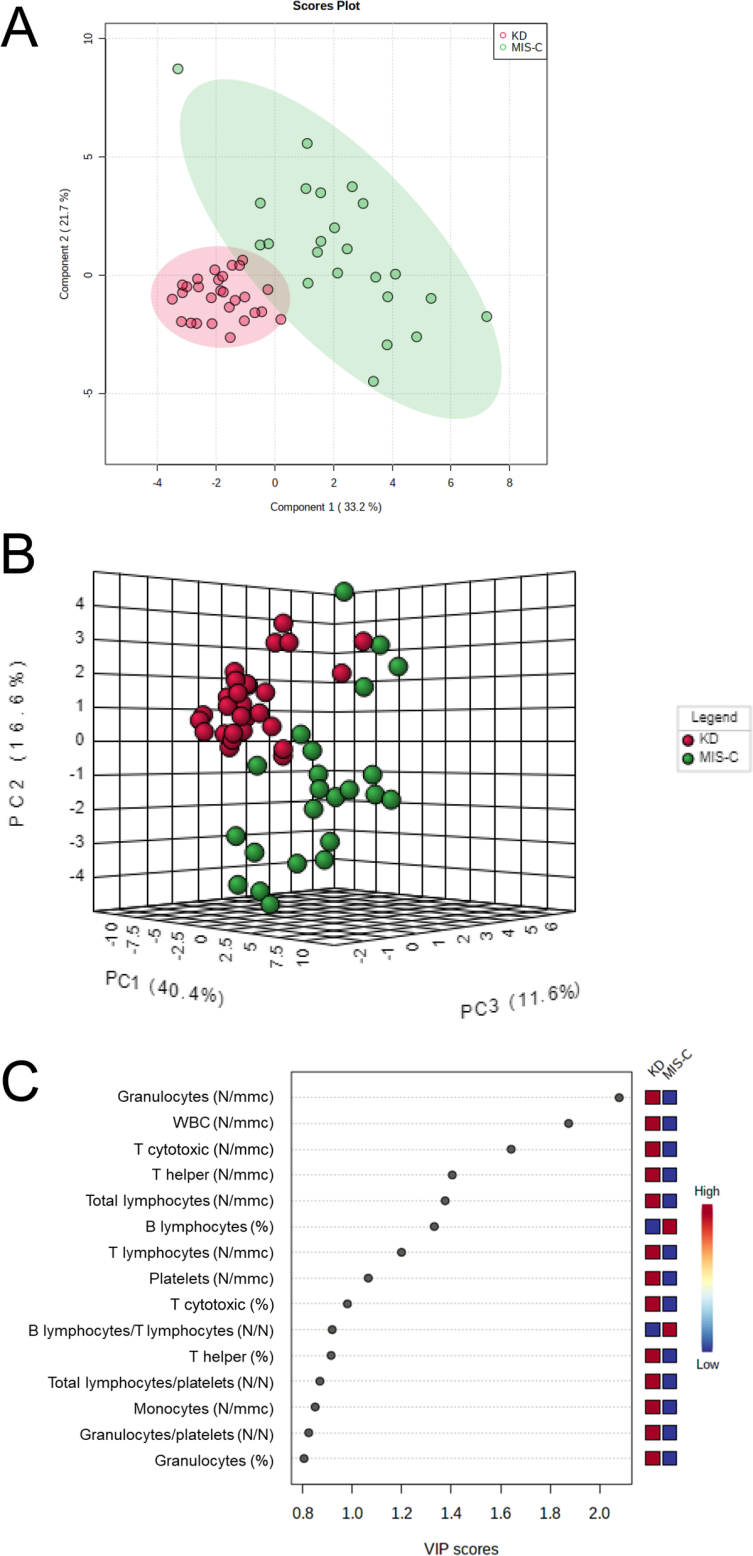
PLS-DA analysis discriminating KD and MIS-C groups. **A**: 2D score plot; **B**: 3D score plot; **C**: VIP score of the first 15 features. KD: Kawasaki disease; MIS-C: multisystem inflammatory syndrome in children; PC: principal component; PLS-DA: partial least-squares discriminant analysis

**Fig. 3:**
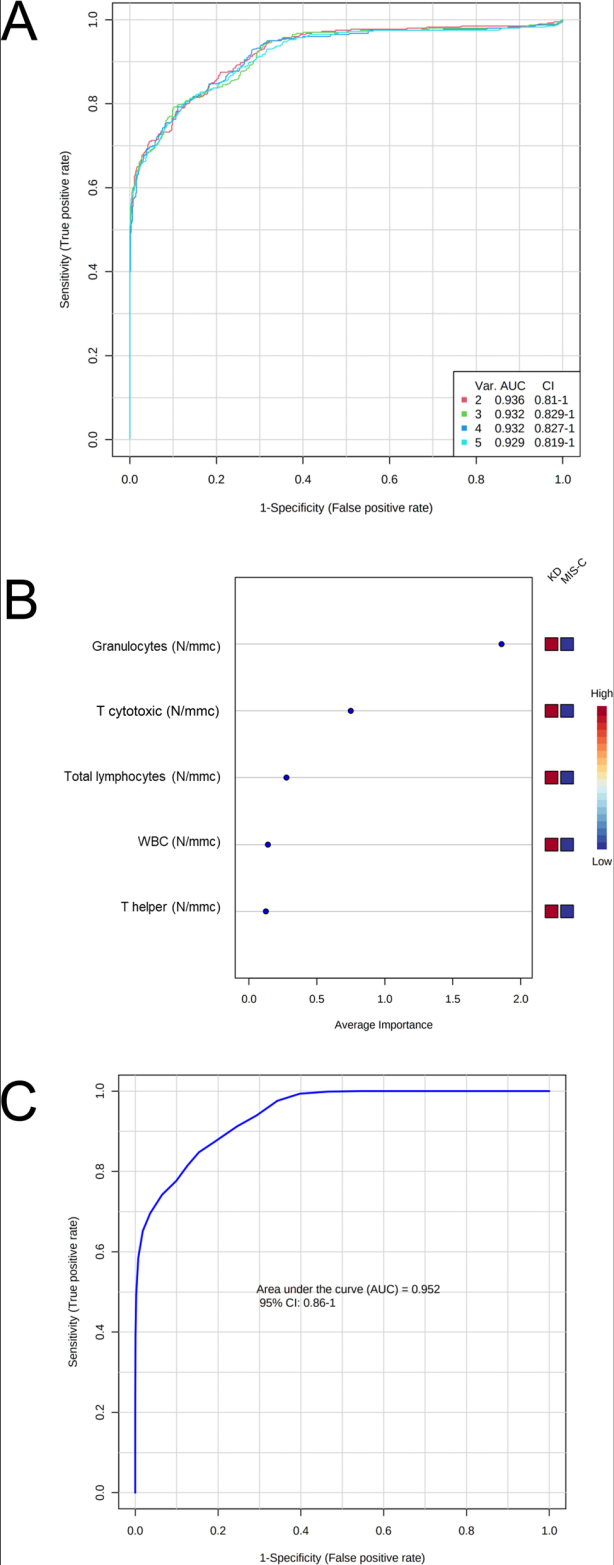
Multivariate ROC curve analyses. **A**: comparison of ROC curves to discriminate MIS-C from KD patients using from 2 to 5 variables (Explorer); **B**: rank features for average importance; **C**: ROC curve Tester using granulocyte number and T cytotoxic lymphocyte number as features. AUC: area under the ROC curve; KD: Kawasaki disease; MIS-C: multisystem inflammatory syndrome in children; ROC: receiver operating characteristic; WBC: white blood cells

We applied PCA, PLS-DA and ROC analyses on the first 23 MIS-C patients that were admitted between June and November 2021 and 28 KD patients. The subsequent 23 MIS-C patients that were admitted between December 2021 and April 2022 were tested to predict the class as well as to validate the model. PCA 2D score plot showed that KD patients clustered in a zone overlapping the zone of patients with MIS-C (Figure S6-A, Additional file 1). While, the PCA synchronized 3D plot (Figure S6-B, Additional file 1) showed that the first (PC1), the second (PC2) and the third (PC3) component separated MIS-C from KD patients. The PLS-DA 2D score plot showed that the first and the second components (PC1 and PC2) separate MIS-C from KD patients and explain the 54.9% of model variance (Fig. 2A). From this analysis we observed that only two KD patients overlapped the cluster of MIS-C group. The MIS-C and KD groups clustered better in the PLS-DA synchronized 3D plot (Fig. 2B). Figure 2C showed the VIP score of the first 15 variables in the PLS-DA model. Then, we performed a ROC Explorer analysis using the first 5 variables with a VIP score higher than 1.4, i.e., granulocytes (N/mmc), WBC (N/mmc), T cytotoxic (N/mmc), T helper (N/mmc), and total lymphocytes (N/mmc). Figure 3A shows the ROC curves based on the cross-validation performance. The area under the ROC curve (AUC) Explorer ranged from 0.929 to 0.936 for the 5 features-model and the 2 features-model, respectively. According to the criteria of Jones and Athanasiou [18], the 2 features-model shows a “very good” AUC. The rank features shows that the granulocyte number together with T cytotoxic lymphocyte number had the higher average importance in the 2 features-model (Fig. 3B). The predictive accuracies ranged from 82.8% for 5 feature-model to 84% for 2 feature-model (Figure S7, Additional file 1). Figure S8 (Additional file 1) shows the predicted class probabilities (average of the cross-validation) for the first 23 MIS-C patients and the 28 KD patients using the best classifier, i.e., the 2 feature-model, with a specificity of 82.1% and a sensitivity of 86.9%.

Finally, we performed a ROC Tester analysis using granulocyte number and T cytotoxic lymphocyte number as features. The AUC of the ROC curve Tester was 0.952 (Fig. 3C). The average accuracy based on 100 cross validations was 85.2%. The predicted class probabilities are depicted in Figure S9 (Additional file 1) with a specificity of 85.7% and a sensitivity of 86.9%. This biomarker model has been used to predict the class of the subsequent 23 MIS-C patients, as independent group. As reported in Table S1, the model classified 19/23 new samples as MIS-C with a probability average of 0.93% (range: 0.62%—0.99%) and a diagnostic sensitivity of 82.6%.

## Discussion

The analysis of WBC in patients with KD and in patients with MIS-C revealed several differences between the two diseases. In fact, patients with KD had a significantly higher number of leukocytes and platelets; furthermore, patients with KD had a significantly higher number of granulocytes, lymphocytes, and monocytes, while patients with MIS-C had a lymphopenia with a significant reduction of the number of B, T, T helper and T cytotoxic lymphocytes. The neutrophilia was previously described only in patients with KD [16], while our data indicate that an increase of the absolute number of circulating neutrophils is present in both the diseases, although it is significantly more relevant in patients with KD. A normal or a higher number of platelets in patients with KD and the thrombocytopenia in patients with MIS-C have been found also by other authors [19]. They suggested that the platelet count may discriminate between the two entities, while our data exclude this possibility for the large overlapping of the data between patients with MIS-C and KD. These authors suggested that the thrombocythemia, as like as the increased number of neutrophils and monocytes observed in patients with KD before the starting of therapies, would be due to the activation of inflammatory cells and to the subsequent recruitment of thrombocytes. We reinforce such hypothesis since all patients with KD from the present study were analyzed at hospital admission, before starting therapy [19]. While, the thrombocytopenia observed in patients with MIS-C is not due to the viral entry in platelets with the subsequent damage, as we recently demonstrated in patients with acute COVID [20]. Likely, it could be due to the suppression of bone marrow and to platelet activation and consumption [19]. Such mechanism can also explain the relevant lymphopenia that we and others observed in patients with MIS-C. In fact, the main difference we find between KD and MIS-C patients is the significant reduction of B, T, helper and cytotoxic populations that we found in these latter and not in patients with KD. These data is in agreement with a previous study that described a significant T lymphopenia (in our case we found also a significant reduction of B lymphocytes) during the acute phase of MIS-C with the subsequent reversion during resolution, again similarly to what we observed in adult patients with severe COVID, that had a significant B and T cell lymphopenia [12] reverted after one week of hospitalization [13]. While, differently from our results, a previous study described the alteration of cytotoxic lymphocytes in KD [16] and another reported a suppression of peripheral activity but not a reduction of the number of CD8 in 7 patients with KD [14].

Finally, the WBC picture observed in patients with MIS-C resembles that observed in adult severe COVID patients with an hyperinflammation due to the cytokine storm, mainly IL-6. However, the increase of IL-10 and IFN observed in all patients with MIS-C and only in few adult patients with severe COVID infection [12], suggests that the origin of MIS-C would be a post-infectious, immunologically “amplified” sequel of COVID [5] in children with a previous mild or asymptomatic acute infection by SARS-CoV-2.

In fact, a study of 95 patients with MIS-C demonstrated that all were serologically positive to SARS-CoV-2, but only a half were still positive to SARS-CoV-2 RT-PCR assay and only 24 referred a COVID compatible illness in the previous weeks [21]. On the other hand, even in hospitalized children with acute COVID infection no alterations in lymphocytes and in the main subpopulations were observed [22]. The less severe expression of COVID in childhood is due to the lower expression of Sars-Cov-2 receptors either in the respiratory and in the gastrointestinal tract [23], to a more robust humoral response [16] and to lower levels of acute phase reactants, among which IL-6 [22] that in adult patients with COVID was strictly correlated with lymphopenia [12].

The picture that we observed in patients with KD is the typical response of the immune system to systemic inflammation [11] with the increase of neutrophils, monocytes and platelets, in agreement with the pathogenesis of a systemic vasculitis with no involvement of lymphocyte subpopulations.

The differences of WBC that we found in patients with KD and MIS-C may have an impact on the diagnosis of two complex disorders for which no diagnostic markers are still available, considered that two-third of patients with MIS-C require critical care support [24] and KD patients need to be early treated to prevent severe cardiological complications [16]. Our data indicate that the granulocyte/lymphocyte ratio is a rapid tool to identify a MIS-C or KD with a high diagnostic sensitivity and specificity. The increase of such ratio was already reported in patients with refractory KD [10] and in patients with a higher risk of KD complications [11, 25]. Our data indicate that the ratio is altered and permits to identify also patients with MIS-C. We already studied the granulocyte/lymphocyte ratio in adult patients with COVID reporting that the ratio would be influenced by anti-inflammatory therapies [26], thus, we suggest its use at hospital admission before starting therapy. Furthermore, given the clinical homology between MIS-C and KD, it would be relevant to discriminate between the two disorders, also because the therapy for MIS-C was originally borrowed from KD with the use of IV Ig, but unlike KD patients, 30–80% of patients with MIS-C do not respond to Ig requiring other drugs, mainly immunomodulators [16]. None of the parameters alone is efficient in discriminating between the two diseases, but our data demonstrate that the granulocyte number together with T cytotoxic lymphocyte number allow to discriminate the two diseases with a high diagnostic efficiency.

The main limitations of our study are the low number of patients with KD and MIS-C analyzed so far and the lack of new patients without a clear diagnosis of MIS-C or KD for extra validation purpose, thus larger studies are necessary to confirm our data. The low number of patients depends on the one hand on the rarity of the two diseases [2, 4] and on the other hand on undiagnosed cases due to an unclear clinical phenotype.

## Conclusions

The present study contributes to conclude that the pathogenesis of MIS-C and KD seem to be different. Moreover, this study represents a tool for a rapid diagnostic contribution. The careful analysis of WBC and of the main lymphocyte subpopulations at admission may help to early diagnose MIS-C and KD and to discriminate between the two diseases.

## Supplementary Information

**Additional file 1: Figure S1.** Gating strategy. A)Lymphocyte cells were gated by using CD45, and this gate was used to identifythe T helper (TH) cells (CD3+ and CD4+) and T cytotoxic (CD3+ and CD8+)lymphocytes. B) CD19 was used to identify B cells. **Figure S2.** Comparison of white blood cells (WBC) andplatelets number in controls (*n*=70), MIS-C (*n*=46) and KD (*n*=28) patients athospital admission. *p < 0.01; ***p < 0.0001. KD: Kawasaki disease;MIS-C: multisystem inflammatory syndrome in children. **Figure S3.** Comparison of granulocytes, lymphocytes andmonocytes, as percentage and absolute number, in controls (*n*=70), MIS-C (*n*=46)and KD (*n*=28) patients at hospital admission. *p < 0.01; **p < 0.001;***p < 0.0001. KD: Kawasaki disease; MIS-C: multisystem inflammatorysyndrome in children. **Figure S4.**Comparison of T, T helper and T cytotoxic lymphocytes, as percentage andabsolute number, in controls (*n*=70), MIS-C (*n*=46) and KD (*n*=28) patients at hospitaladmission. **p < 0.001; ***p < 0.0001. KD: Kawasaki disease; MIS-C:multisystem inflammatory syndrome in children. **Figure S5.** Comparison ofB lymphocytes, as percentage and absolute number, in controls (*n*=70), MIS-C(*n*=46) and KD (*n*=28) patients at hospital admission. ***p < 0.0001. KD:Kawasaki disease; MIS-C: multisystem inflammatory syndrome in children. **Figure S6.** PCA analysisdiscriminating KD and MIS-C groups. A: 2D score plot; B: 3D score plot; KD:Kawasaki disease; MIS-C: multisystem inflammatory syndrome in children; PC:principal component; PCA: principal component analysis.** Figure S7**.Predictive accuracies of models from 2 to 5 variables of multivariate ROC curvebased exploratory analysis. ROC: receiver operating characteristic.

## Data Availability

All data used during the study are available from the corresponding author by request.

## Abbreviations

KD: Kawasaki disease
MIS-C: Multisystem inflammatory syndrome in children
PCA: Principal component analysis
PLS-DA: Partial least-squares discriminant analysis
ROC: Receiver operating characteristic
WBC: White blood cells
