# Supplementary material for: Immunophenotyping of peripheral blood cells allows to discriminate MIS-C and Kawasaki disease
Source: Transl Med Commun. 2022 Sep 4;7(1):22. doi: 10.1186/s41231-022-00128-2 (PMC9440857; doi:10.1186/s41231-022-00128-2)
Supplement: Supplementary file 1 — Additional file 1: Figure S1. Gating strategy. A)Lymphocyte cells were gated by using CD45, and this gate was used to identifythe T helper (TH) cells (CD3+ and CD4+) and T cytotoxic (CD3+ and CD8+)lymphocytes. B) CD19 was used to identify B cells. Figure S2. Comparison of white blood cells (WBC) andplatelets number in controls (n=70), MIS-C (n=46) and KD (n=28) patients athospital admission. *p < 0.01; ***p < 0.0001. KD: Kawasaki disease;MIS-C: multisystem inflammatory syndrome in children. Figure S3. Comparison of granulocytes, lymphocytes andmonocytes, as percentage and absolute number, in controls (n=70), MIS-C (n=46)and KD (n=28) patients at hospital admission. *p < 0.01; **p < 0.001;***p < 0.0001. KD: Kawasaki disease; MIS-C: multisystem inflammatorysyndrome in children. Figure S4.Comparison of T, T helper and T cytotoxic lymphocytes, as percentage andabsolute number, in controls (n=70), MIS-C (n=46) and KD (n=28) patients at hospitaladmission. **p < 0.001; ***p < 0.0001. KD: Kawasaki disease; MIS-C:multisystem inflammatory syndrome in children. Figure S5. Comparison ofB lymphocytes, as percentage and absolute number, in controls (n=70), MIS-C(n=46) and KD (n=28) patients at hospital admission. ***p < 0.0001. KD:Kawasaki disease; MIS-C: multisystem inflammatory syndrome in children. Figure S6. PCA analysisdiscriminating KD and MIS-C groups. A: 2D score plot; B: 3D score plot; KD:Kawasaki disease; MIS-C: multisystem inflammatory syndrome in children; PC:principal component; PCA: principal component analysis. Figure S7.Predictive accuracies of models from 2 to 5 variables of multivariate ROC curvebased exploratory analysis. ROC: receiver operating characteristic. [file 41231_2022_128_MOESM1_ESM.pdf]

A

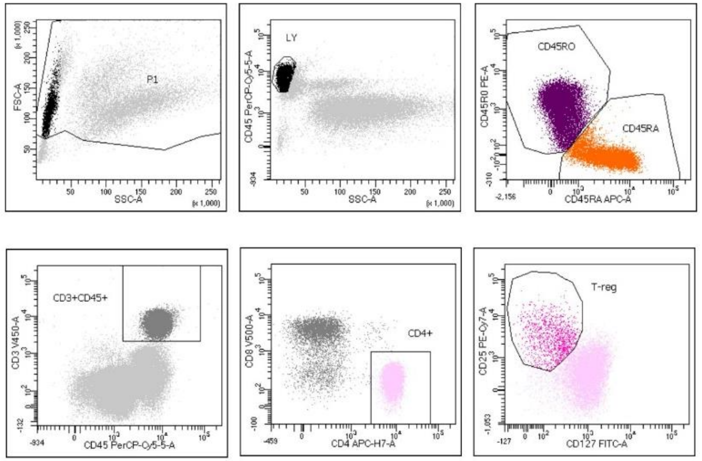

B

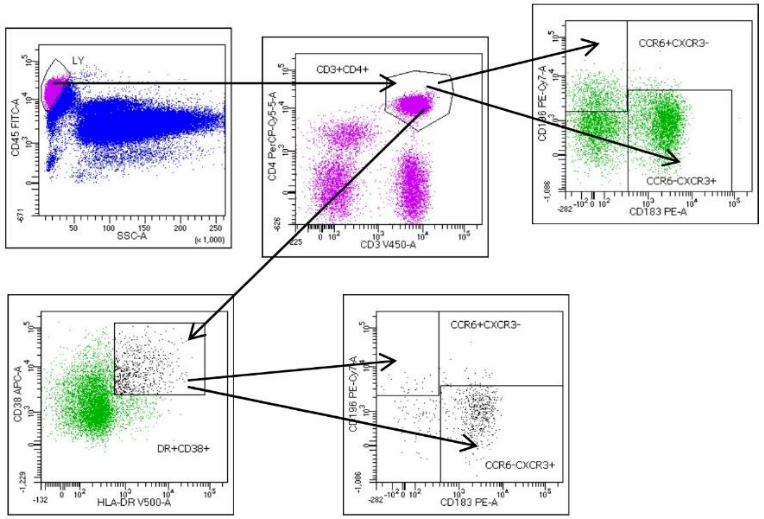

Figure S1. Gating strategy. A) Lymphocyte cells were gated by using CD45, and this gate was used to identify the T helper (TH) cells (CD3+ and CD4+) and T cytotoxic (CD3+ and CD8+) lymphocytes. B) CD19 was used to identify B cells.

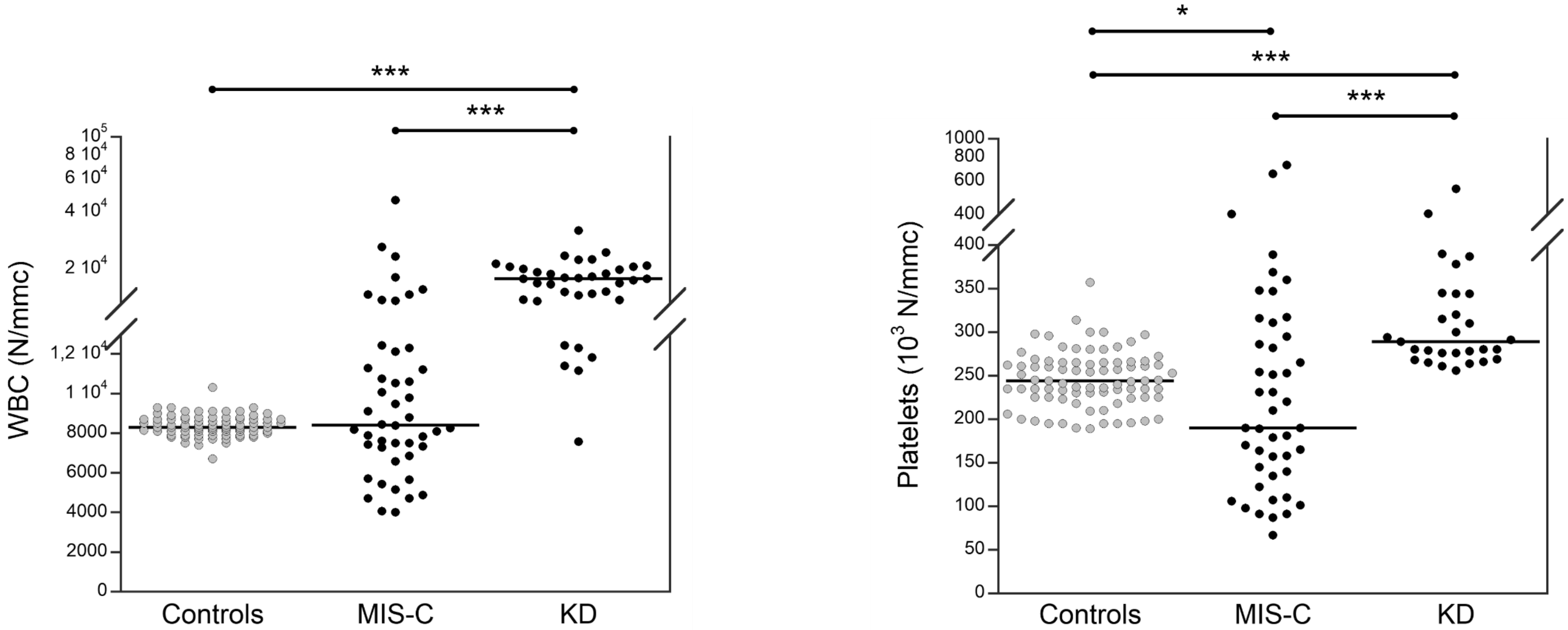

Figure S2. Comparison of white blood cells (WBC) and platelets number in controls (n=70), MIS-C (n=46) and KD (n=28) patients at hospital admission. *p < 0.01; ***p < 0.0001. KD: Kawasaki disease; MIS-C: multisystem inflammatory syndrome in children.

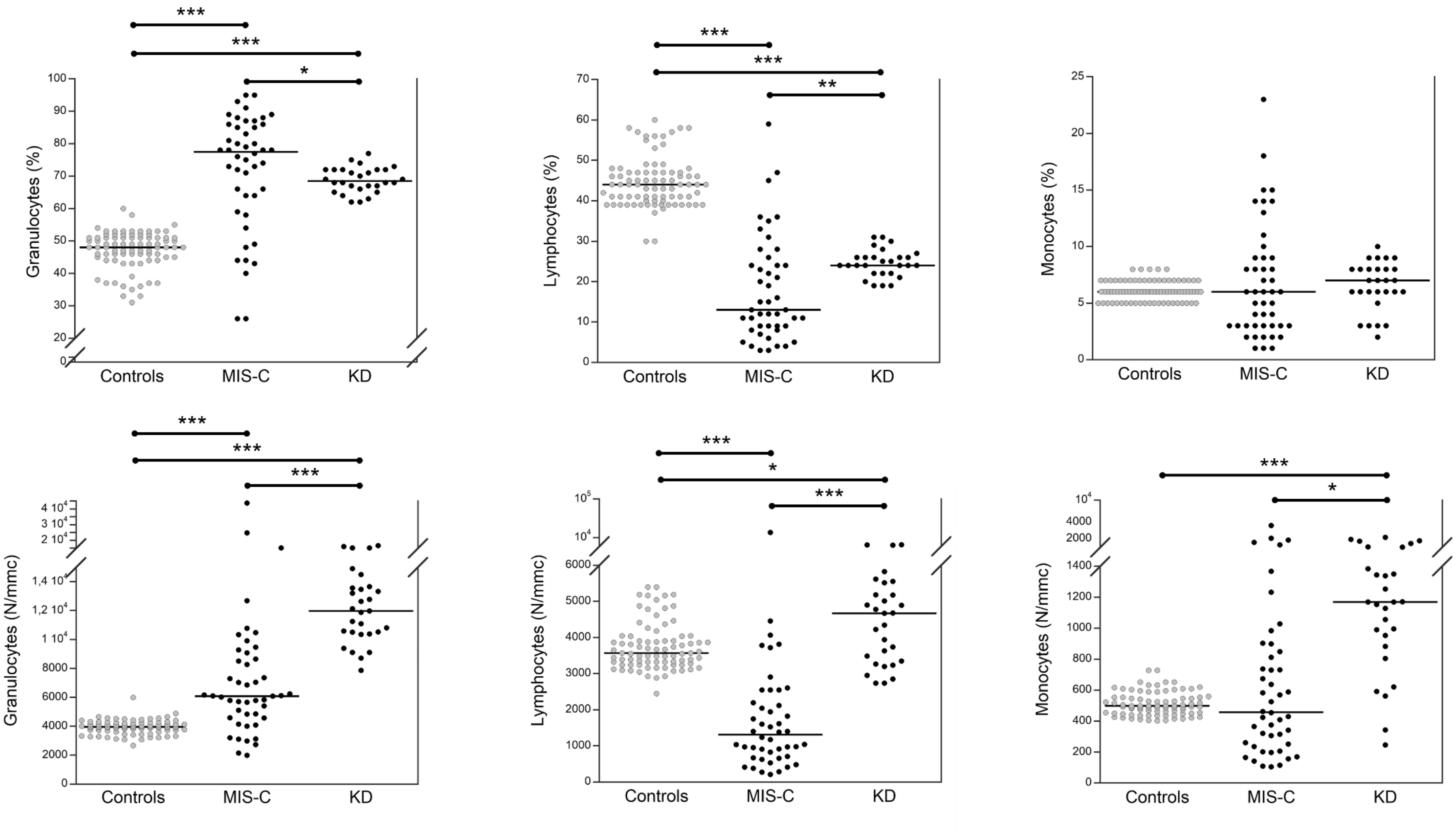

Figure S3. Comparison of granulocytes, lymphocytes and monocytes, as percentage and absolute number, in controls (n=70), MIS-C (n=46) and KD (n=28) patients at hospital admission. *p < 0.01; **p < 0.001; ***p < 0.0001. KD: Kawasaki disease; MIS-C: multisystem inflammatory syndrome in children.

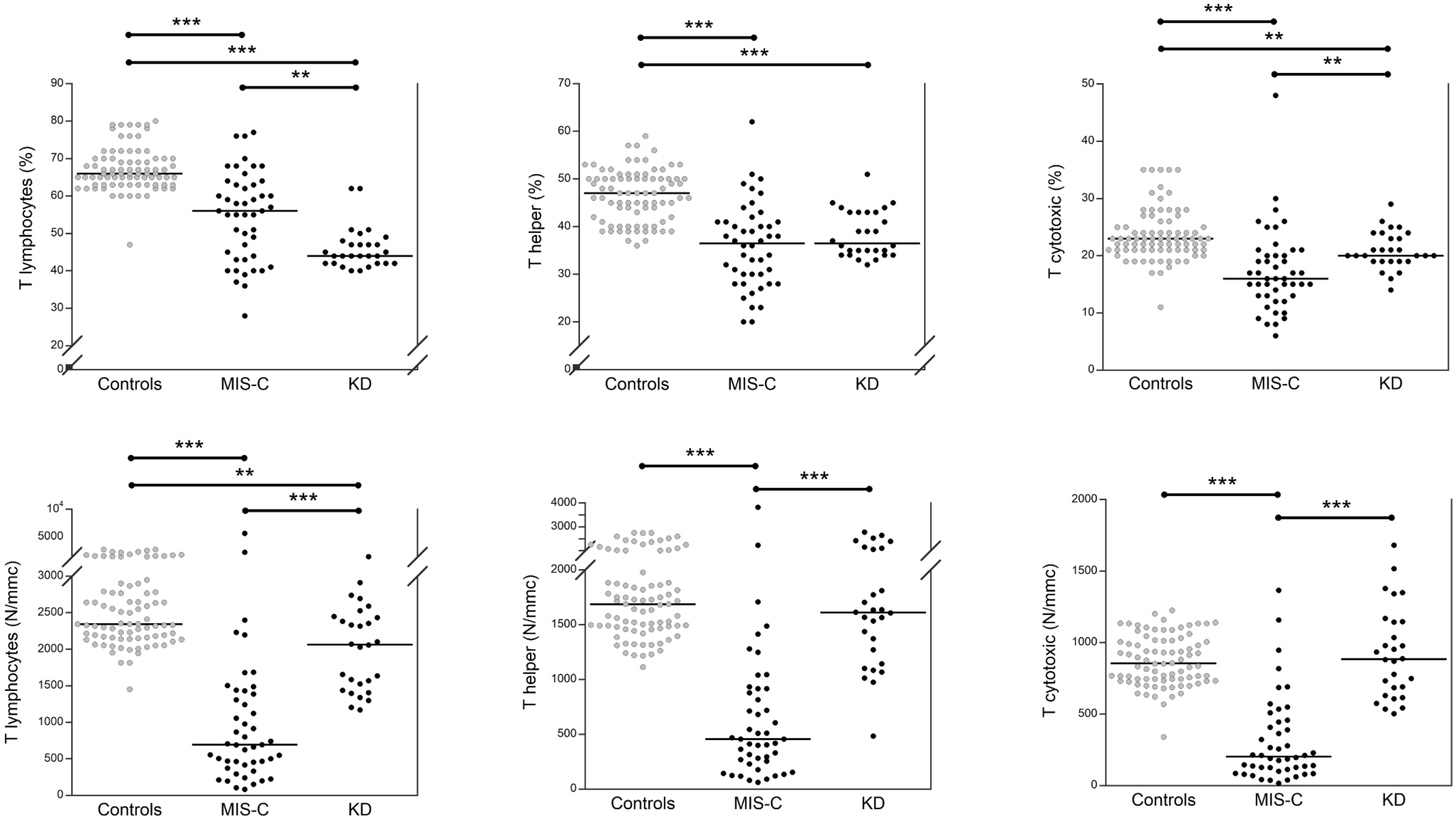

Figure S4. Comparison of T, T helper and T cytotoxic lymphocytes, as percentage and absolute number, in controls (n=70), MIS-C (n=46) and KD (n=28) patients at hospital admission. **p < 0.001; ***p < 0.0001. KD: Kawasaki disease; MIS-C: multisystem inflammatory syndrome in children.

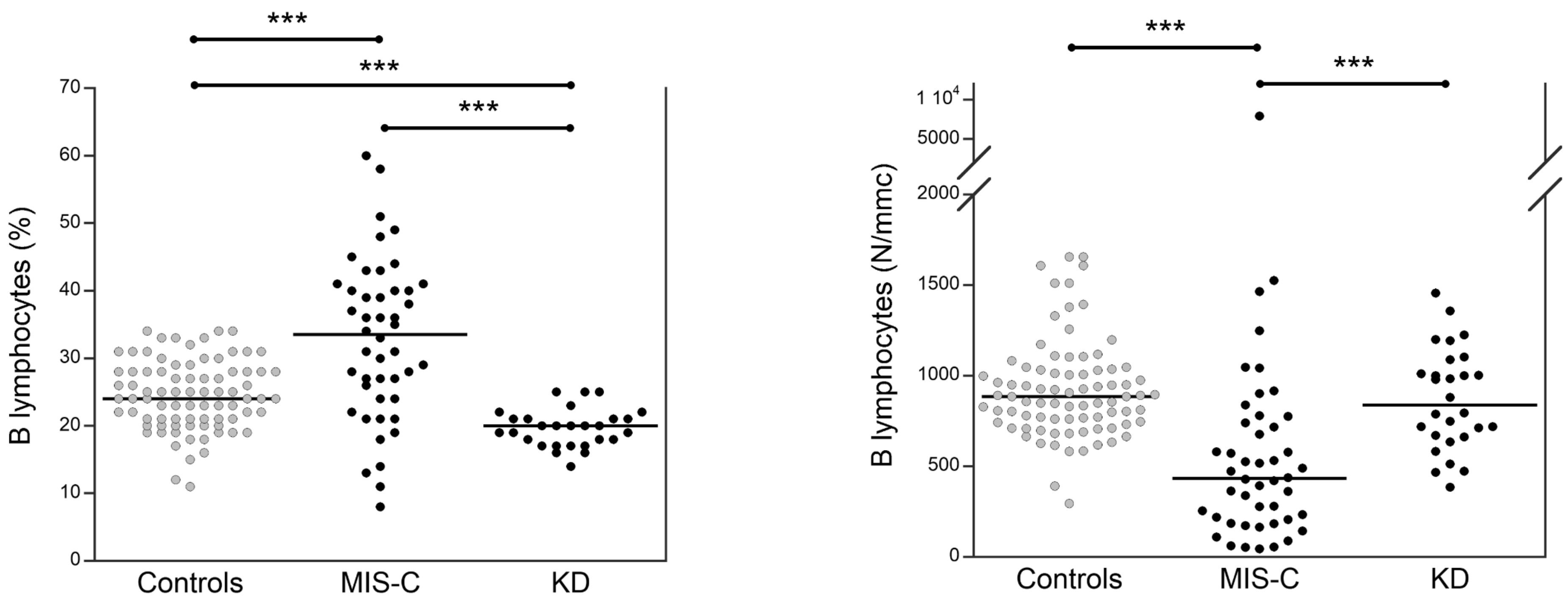

Figure S5. Comparison of B lymphocytes, as percentage and absolute number, in controls (n=70), MIS-C (n=46) and KD (n=28) patients at hospital admission. ***p < 0.0001. KD: Kawasaki disease; MIS-C: multisystem inflammatory syndrome in children.

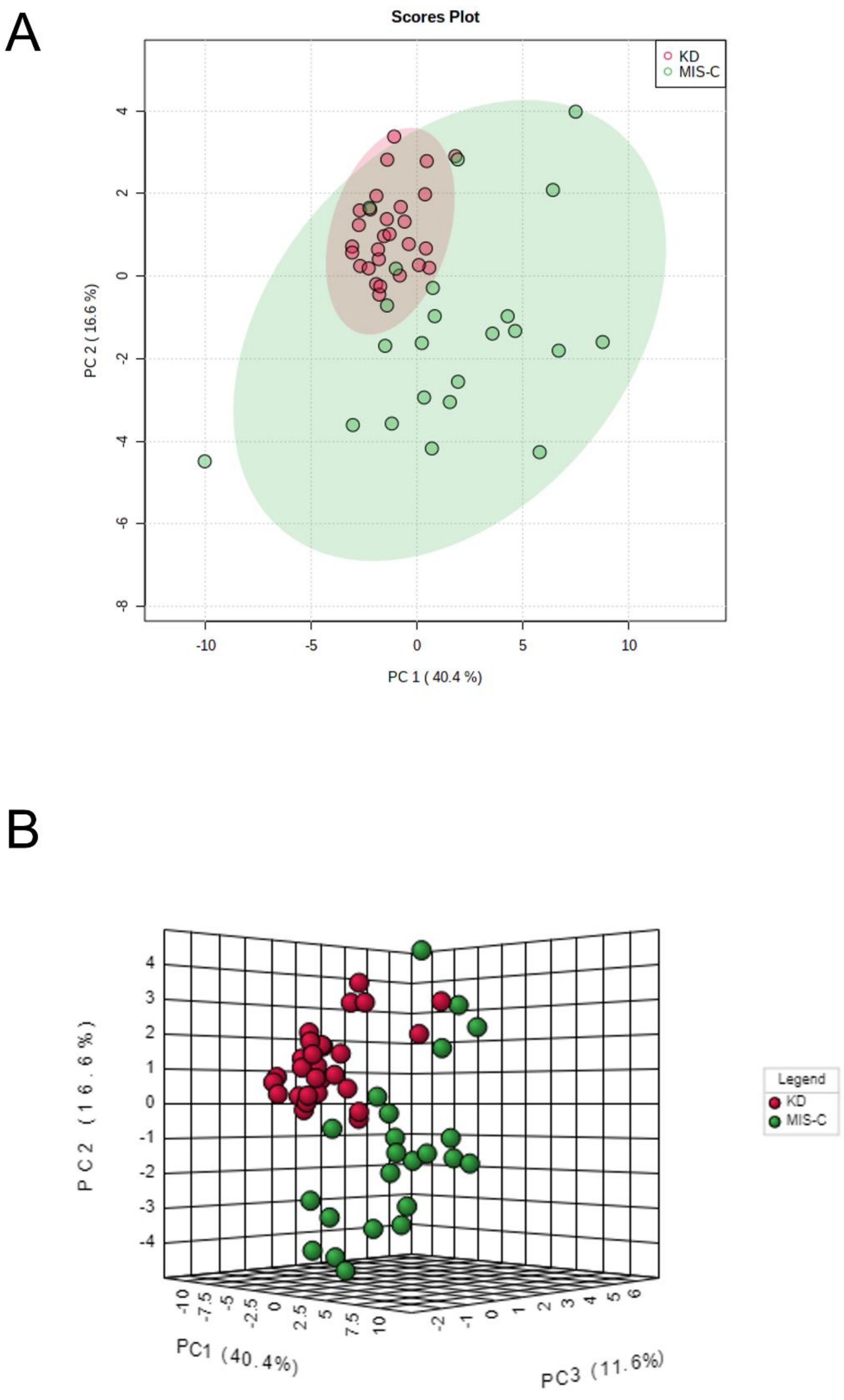

Figure S6. PCA analysis discriminating KD and MIS-C groups. A: 2D score plot; B: 3D score plot; KD: Kawasaki disease; MIS-C: multisystem inflammatory syndrome in children; PC: principal component; PCA: principal component analysis.

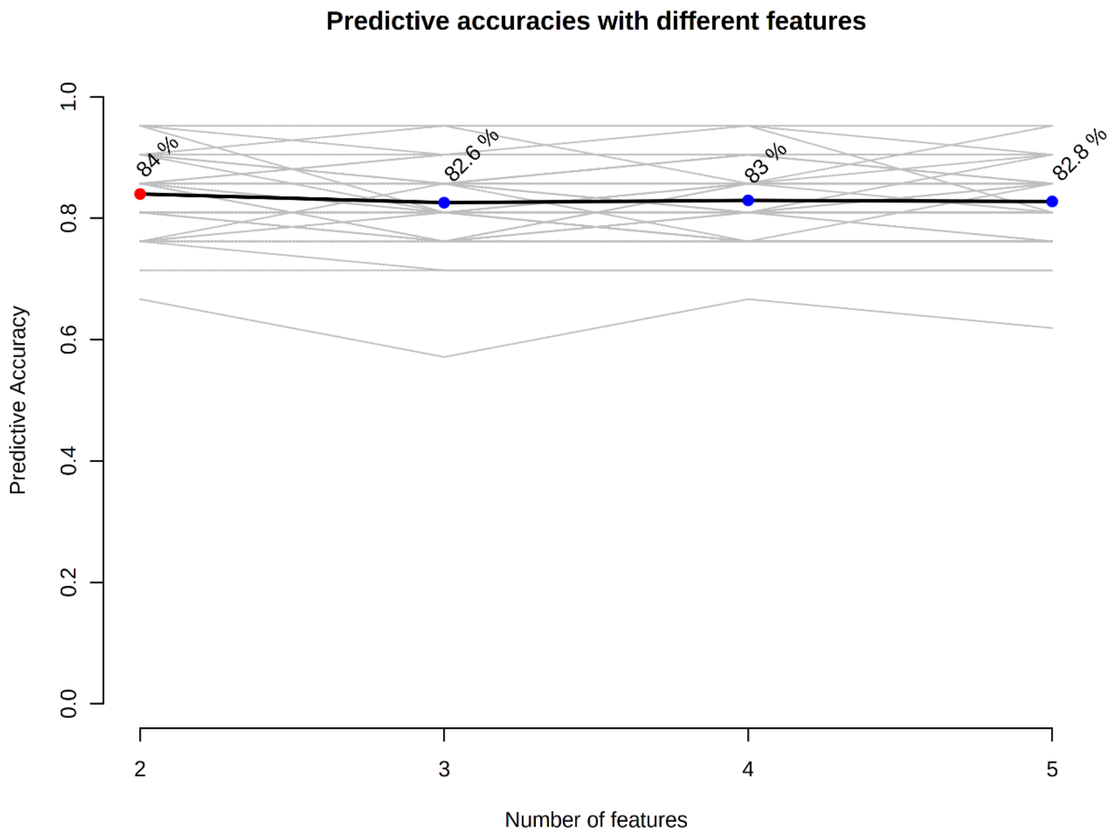

Figure S7. Predictive accuracies of models from 2 to 5 variables of multivariate ROC curve based exploratory analysis. ROC: receiver operating characteristic.

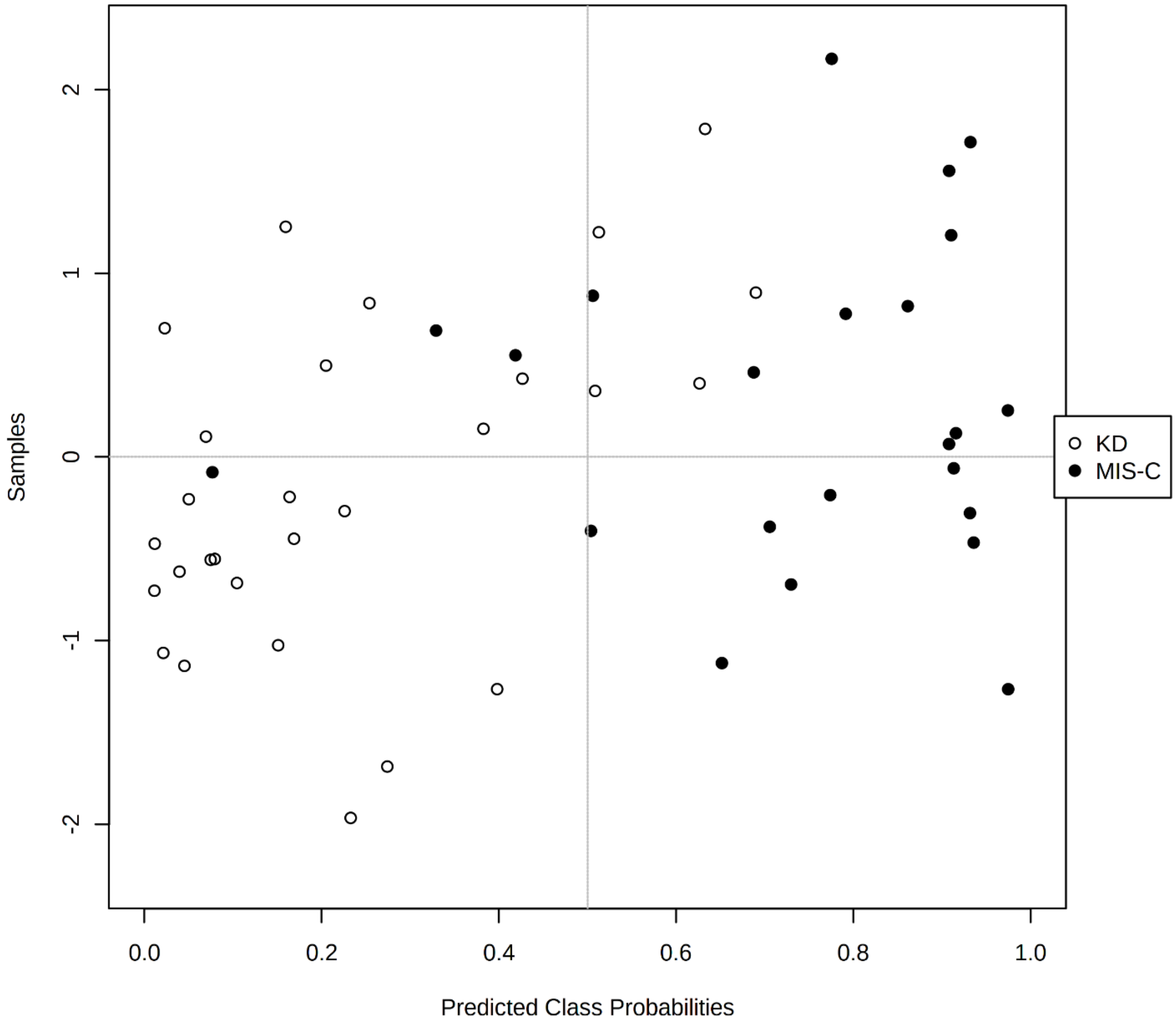

Figure S8. Predicted class probabilities (average of the cross-validation) for the first 23 MIS-C patients and the 28 KD patients using the 2 feature-model of the ROC curve Explorer.

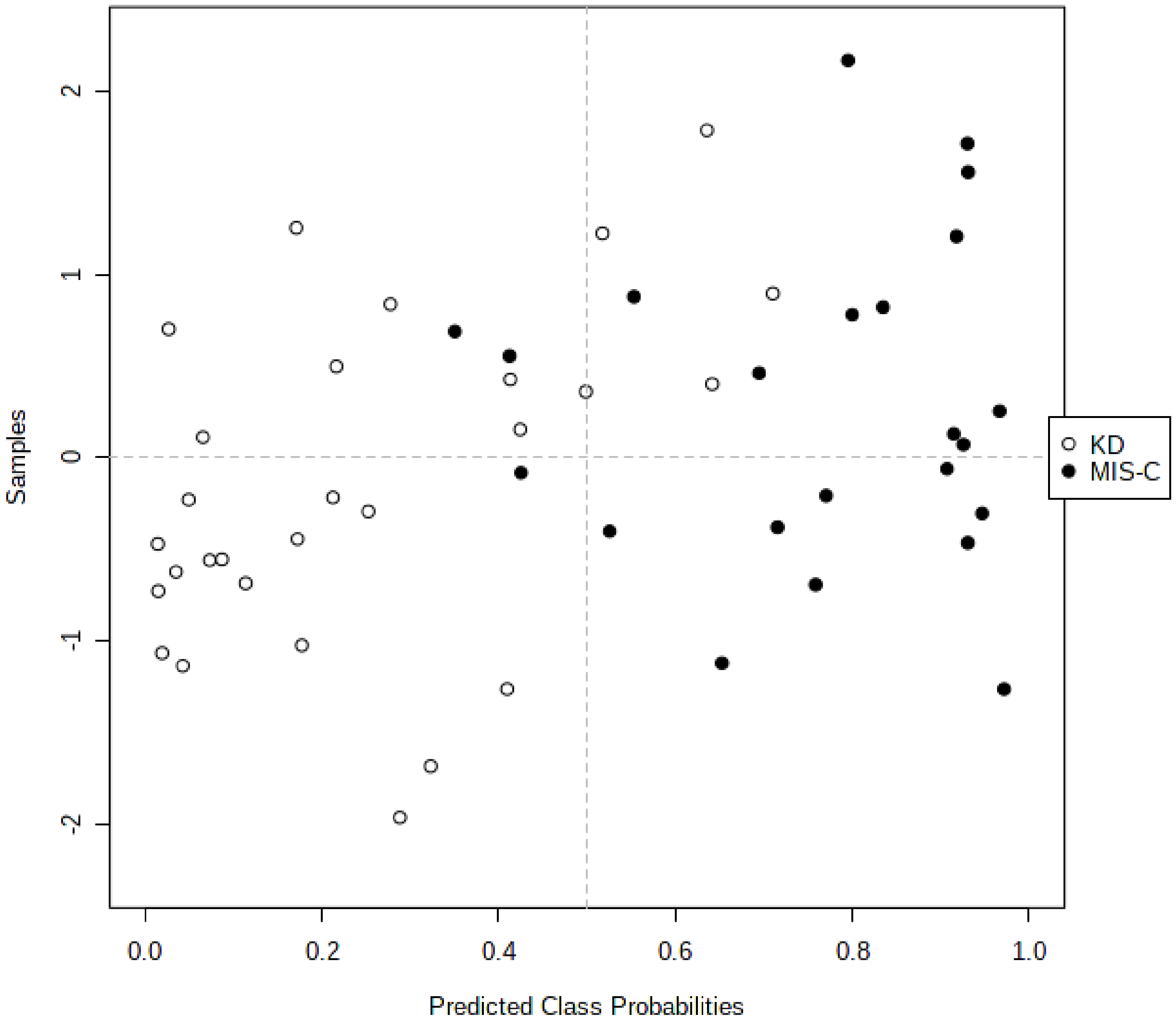

Figure S9. Predicted class probabilities (average of the cross-validation) for the first 23 MIS-C patients and the 28 KD patients using the ROC curve Tester.

Table S1. New sample prediction

Sample	Probability	Class
# 24	0.92275	MIS-C
# 25	0.96282	MIS-C
# 26	0.75594	MIS-C
# 27	0.94463	MIS-C
# 28	0.9202	MIS-C
# 29	0.98366	MIS-C
# 30	0.97782	MIS-C
# 31	1.0	KD
# 32	0.96591	MIS-C
# 33	0.97673	MIS-C
# 34	0.9378	MIS-C
# 35	0.61345	KD
# 36	0.62605	MIS-C
# 37	0.99211	MIS-C
# 38	0.98241	MIS-C
# 39	0.96064	MIS-C
# 40	0.92892	MIS-C
# 41	0.8879	KD
# 42	0.97463	MIS-C
# 43	0.99924	KD
# 44	0.99462	MIS-C
# 45	0.9818	MIS-C
# 46	0.95867	MIS-C

KD: Kawasaki disease; MIS-C: multisystem inflammatory syndrome in children.
